# Secondary Prophylaxis With Bioequivalent Generic Letermovir in Haploidentical Hematopoietic Stem Cell Transplantation: A Case Report

**DOI:** 10.7759/cureus.101126

**Published:** 2026-01-08

**Authors:** Abhijit Baheti, Suyash Bharat

**Affiliations:** 1 Hematology, Deenanath Mangeshkar Hospital, Pune, IND; 2 Medical Affairs, Zydus Lifesciences, Ahmedabad, IND

**Keywords:** cytomegalovirus reactivation, graft rejection, haploidentical haematopoietic stem cell transplantation, letermovir, prophylaxis

## Abstract

Cytomegalovirus (CMV) reactivation poses a significant risk post-allogeneic hematopoietic stem cell transplantation (HSCT), particularly in high-risk settings. This report details the clinical course of a relapsed acute myeloid leukemia patient who underwent haploidentical HSCT following graft failure linked to CMV viremia. Letermovir prophylaxis effectively suppressed CMV but was discontinued on day +60, following which the patient experienced transient reactivation. The episode was successfully managed in the outpatient setting with oral valganciclovir. This case highlights the importance of sustained CMV prophylaxis and the role of accessible antiviral strategies in optimizing transplant outcomes.

## Introduction

Cytomegalovirus (CMV) remains a significant cause of morbidity and mortality after allogeneic hematopoietic stem cell transplantation (HSCT) [1]. CMV reactivation occurs in a substantial proportion of patients within the first year post-transplant, with incidence rates varying based on donor type, conditioning regimen, and degree of immunosuppression. Recent multicenter data report one-year reactivation rates as high as 62% in haploidentical transplants, compared with 39% and 44% in matched-sibling and unrelated-donor settings, respectively [2].

In the absence of effective prophylaxis, CMV reactivation contributes significantly to non-relapse mortality, primarily via opportunistic infections and end-organ complications [3]. Preemptive treatment strategies, while effective, often depend on intensive virologic monitoring and involve agents such as ganciclovir or foscarnet, drugs limited by their hematologic and renal toxicities [4]. These limitations are particularly pronounced in the early post-transplant period, when marrow recovery is incomplete, and patients are most vulnerable.

In addition to established prophylactic and preemptive strategies, newer antiviral agents such as maribavir are now available for the management of refractory or resistant CMV infection in transplant recipients. One such is letermovir. It is a selective inhibitor of CMV deoxyribonucleic acid (DNA) terminase complexes, representing a shift toward prophylaxis in high-risk settings. Its favorable safety profile, particularly the lack of hematologic toxicity, supports its use during periods of marrow vulnerability [5]. While large-scale trials have demonstrated letermovir’s efficacy in preventing CMV reactivation, optimal duration and accessibility remain active areas of investigation [6,7]. This case highlights the clinical course of action for a high-risk transplant recipient in whom bioequivalent generic letermovir (Anvino) secondary prophylaxis effectively prevented early CMV reactivation and explores the virologic and hematologic outcomes following premature discontinuation and subsequent preemptive therapy.

## Case presentation

A 38-year-old male with relapsed acute myeloid leukemia (AML) was admitted on 26/06/2024 to a tertiary care transplant center for planned allogeneic HSCT after achieving remission. At the time of admission, the patient was clinically stable, afebrile, hemodynamically stable, and functionally independent, with no evidence of active infection or organ dysfunction. A matched unrelated donor (MUD) transplant was performed following a standard myeloablative conditioning regimen. Post-engraftment, routine quantitative CMV DNA polymerase chain reaction (PCR) testing revealed CMV viremia, which coincided with a progressive decline in blood counts. On August 9, 2024, the patient had Hb 9.9 g/dL, WBC 0.11 × 10⁹/L with neutrophils 3% (ANC ≈ 0.003 × 10⁹/L), and platelets 5 × 10⁹/L, fulfilling criteria for severe pancytopenia; bone marrow aspirate and trephine were markedly hypocellular with myeloid and megakaryocytic suppression and no excess blasts, confirming marrow hypoplasia. On day +28, the patient was diagnosed with marrow hypoplasia, indicative of graft rejection, as evidenced by the absence of donor chimerism. CMV viremia was considered a potential contributing factor to the graft failure. The patient subsequently developed severe pancytopenia, requiring transfusion support and broad-spectrum antimicrobials. Due to the absence of a second MUD and the urgency of marrow rescue, the decision was made to proceed with haploidentical HSCT using his human leukocyte antigen (HLA)-partially matched sister as the donor. CMV viremia was actively managed during the peritransplant phase with foscarnet, resulting in a steady decline in CMV viral load to target not detected (TND) levels prior to transplantation.

**Figure 1:**
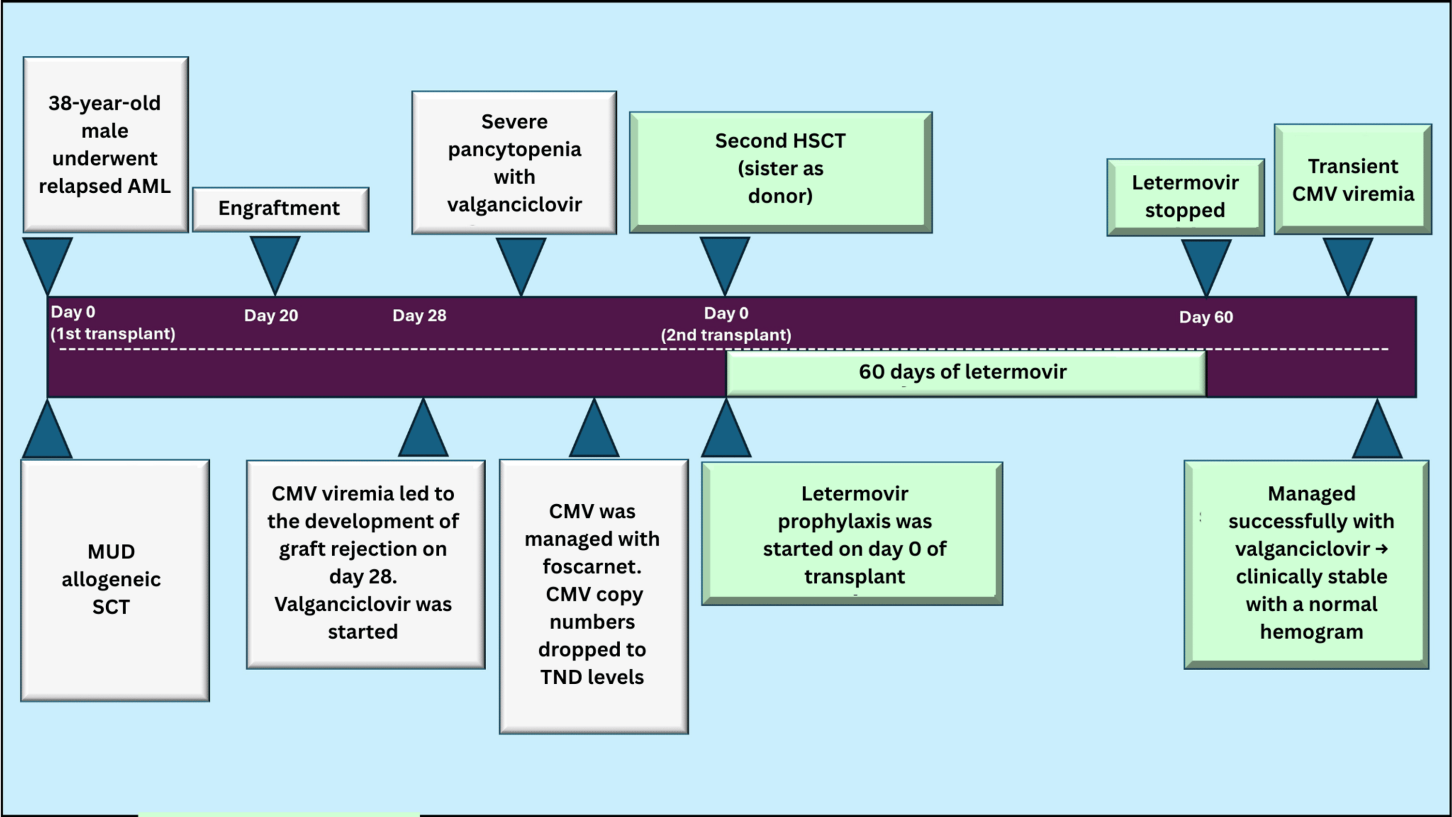
Clinical course of the patient AML: acute myeloid leukemia, CMV: cytomegalovirus, TND: target not detected, HSCT: hematopoietic stem cell transplantation, MUD: matched unrelated donor, SCT: stem cell transplantation

Following haploidentical HSCT, the patient engrafted successfully, and prophylactic letermovir was initiated early in the post-transplant phase. He tolerated the drug well, with no CMV reactivation observed during the first 60 days post-transplant. His complete blood counts improved steadily, and he remained clinically stable. However, letermovir was discontinued on day +60 due to financial constraints. Approximately one week later, the patient developed transient CMV viremia, although he remained clinically well with a normal hemogram. CMV was successfully managed with oral valganciclovir, resulting in viral clearance without further complications. Throughout his post-transplant course, the patient was monitored closely with weekly CMV PCR assays and remained free of further CMV reactivation episodes. He continued to recover hematologically and did not exhibit signs of graft-versus-host disease (GVHD) (Figure 1).

## Discussion

CMV reactivation remains a critical concern in allogeneic stem cell transplantation, particularly in high-risk settings such as haploidentical transplants or after prior graft failure. In the present case, early CMV viremia coincided with graft rejection following a MUD transplant, underscoring the complex interplay between viral replication, immune reconstitution, and hematopoietic recovery.

Following successful engraftment after haploidentical transplantation, early introduction of a CMV terminase complex inhibitor during the post-engraftment period was associated with sustained virologic suppression through day 60 of treatment initiation. During this window, the patient remained clinically stable, with no CMV viremia and progressive hematologic recovery. In clinical trials, similar prophylactic approaches have demonstrated reduced incidence of clinically significant CMV infection and delayed onset of CMV-related complications during the high-risk early post-transplant phase [7]. Within a short interval after letermovir discontinuation, low-level CMV viremia was detected. The temporal proximity between cessation of prophylaxis and viral reactivation is consistent with the known rebound risk in seropositive recipients, particularly when prophylaxis is truncated before day +100 [8]. These observations support a tailored duration of prophylaxis based on individualized risk factors such as donor type, conditioning intensity, and prior CMV dynamics, rather than a fixed timeline.

Notably, the patient did not require hospitalization during letermovir prophylaxis, and subsequent reactivation was managed in the outpatient setting. In the setting of post-prophylaxis viremia, oral valganciclovir was selected for preemptive therapy. The patient tolerated the regimen well, with viral clearance achieved without marrow suppression or neutropenic complications. Valganciclovir, a prodrug of ganciclovir with high oral bioavailability, has demonstrated effective preemptive control of CMV in reduced-intensity conditioning regimens, with comparable pharmacokinetics to intravenous formulations [9]. In patients without gastrointestinal GVHD, oral therapy offers a practical, less invasive approach to viral control without compromising efficacy.

This case showcases the value of early non-myelosuppressive prophylaxis during hematologic vulnerability, combined with strategic preemptive therapy in the event of reactivation. Sustaining such an approach requires careful monitoring and individualized planning to preserve early virologic gains and ensure continued post-transplant stability.

## Conclusions

Bioequivalent generic letermovir prophylaxis provided effective early CMV suppression post-haploidentical SCT without hematologic toxicity. Discontinuation led to transient reactivation, successfully managed with oral valganciclovir. This case underscores the critical need for sustained, risk-adapted CMV prevention strategies extending up to 200 days in high-risk transplant recipients.
